# Exploring a Highly D-Galactose Specific L-Arabinose Isomerase From *Bifidobacterium adolescentis* for D-Tagatose Production

**DOI:** 10.3389/fbioe.2020.00377

**Published:** 2020-04-29

**Authors:** Guoyan Zhang, Yingfeng An, Amreesh Parvez, Hossain M. Zabed, Junhua Yun, Xianghui Qi

**Affiliations:** ^1^School of Food and Biological Engineering, Jiangsu University, Zhenjiang, China; ^2^College of Biosciences and Biotechnology, Shenyang Agricultural University, Shenyang, China

**Keywords:** *Bifidobacterium adolescentis*, L-arabinose isomerase, D-tagatose, sweetener, probiotic bacteria

## Abstract

D-Galactose-specific L-arabinose isomerase (L-AI) would have much potential for the enzymatic conversion of D-Galactose into D-tagatose, while most of the reported L-AIs are L-arabinose specific. This study explored a highly D-Galactose-specific L-AI from *Bifidobacterium adolescentis* (BAAI) for the production of D-tagatose. In the comparative protein-substrate docking for D-Galactose and L-arabinose, BAAI showed higher numbers of hydrogen bonds in D-Galactose-BAAI bonding site than those found in L-arabinose-BAAI bonding site. The activity of BAAI was 24.47 U/mg, and it showed good stability at temperatures up to 65°C and a pH range 6.0–7.5. The *K*_m_, *V*_max_, and *K*_cat_/*K*_m_ of BAAI were found to be 22.4 mM, 489 U/mg and 9.3 mM^–1^ min^–1^, respectively for D-Galactose, while the respective values for L-arabinose were 40.2 mM, 275.1 U/mg, and 8.6 mM^–1^ min^–1^. Enzymatic conversion of D-Galactose into D-tagatose by BAAI showed 56.7% conversion efficiency at 55°C and pH 6.5 after 10 h.

## Introduction

D-tagatose is a rare ketohexose in nature found mostly in gums and lichens, which is widely used as a low caloric sweetener and substitute of sucrose for its 92% sweetness of sucrose in a 10% aqueous solution with only one-third calories of sucrose (Guo et al., 2018). It is also known for its prebiotic action, capability of reducing cholesterol, preventing colon cancer, treatment of type II diabetes, and so on (Lu et al., 2008). It is widely used in many food and pharmaceuticals preparations, including drinks, yogurt, diabetes-specific foods, diet foods, cereals, meat products, candies, cough syrups, anti-adhesives for fixed dentures and oral disinfectants (Kim, 2004; Marylane et al., 2017).

D-tagatose can be produced either by chemical or biological method, where the former is mainly based on the isomerization of D-Galactose into D-tagatose by a chemical catalyst, such as soluble alkali metal salt or an alkaline earth metal salt or potassium aluminate. In spite of its maturity and application on large scale, chemical method has some drawbacks, including production of by-products, requirement of an extensive purification step, and responsibility to environmental pollution (Oh, 2007). In contrast, biological method offers an eco-friendly conversion approach, in which L-arabinose isomerase (L-AI, EC 5.3.1.4) is used for isomerizing D-Galactose into D-tagatose (Zhang et al., 2017, 2019).

To achieve a sustainable bioconversion process for D-tagatose production, utilization of cheap substrate and efficient enzyme are utmost important. In this context, milk industry wastes, particularly whey powder rich in lactose, would be an attractive source of substrates, which provides galactose upon hydrolysis of lactose (Zhang et al., 2020). As attempts to obtain potential source of L-AIs with a high efficiency, more than 30 L-AIs have been reported thus far from various natural or engineered microorganisms, such as *Escherichia coli* (Wu et al., 2020), *Clostridium hylemonae* (Nguyen et al., 2018), *Bacillus subtilis* (Kim et al., 2010), *Bacillus coagulans* (Mei et al., 2016), *Lactobacillus plantarum* (Chouayekh et al., 2007), *Lactobacillus brevis* (Guo et al., 2018), *Geobacillus thermodenitrificans* (Kim and Oh, 2005), *Enterococcus faecium* (Marylane et al., 2017) and *Bifidobacterium longum* (Salonen et al., 2012). However, almost all of these L-AIs showed higher specificity to L-arabinose than the specificity to D-Galactose (Xu et al., 2018). This has indicated that existing L-AIs might not be as efficient for valorizing milk whey powder as desired. Therefore, screening and characterization of highly galactose-specific L-AIs should receive significant research priority. Furthermore, D-tagatose produced from D-Galactose derived from whey powder requires hydrolysis of its lactose, which need to be carried out under acidic condition (Zhan et al., 2014; Zhang et al., 2020).

In this study, we report a highly D-Galactose-specific L-AI from *Bifidobacterium adolescentis* (BAAI) for the first time, which worked under acidic condition. This enzyme was structurally and biochemically characterized after cloning and expressing its relevant gene (*araA)* in *E. coli* BL21(DE3). The purified enzyme was studied to determine the optimum conditions for obtaining its maximum activity and to evaluate its substrate specificities. Subsequently, structural homology and protein-substrate docking of the BAAI were analyzed to explain the basis of substrate specificity. Finally, enzymatic conversion of D-Galactose into D-tagatose was carried out using the purified BAAI for evaluating its conversion efficiency.

## Materials and Methods

### Bacterial Strains and Materials

*B. adolescentis* CICC 6178 strain was obtained from the China Center of Industrial Culture Collection (CICC), Beijing, China. It was cultivated anaerobically in TPY fluid Medium at 37°C without agitation. pANY1 plasmid was obtained from Shenyang Agricultural University, China (Gao et al., 2018; Liu et al., 2018), which was used as the expression vector. *E. coli* BL21(DE3) was obtained from the Stratagene (California, United States). Q5 DNA Polymerase and Restriction Endonucleases were bought from the New England Biolab (NEB) (Beijing, China). ClonExpress^®^ One-Step Cloning Kit, FastPure Plasmid Mini Kit, and FastPure Gel DNA Extraction Mini Kit were purchased from Vazyme (Nanjing, China). Solubilization and Refolding Kit was bought from the TIANDZ (Beijing, China). All other chemicals and reagents were purchased from the Sinopharm (Beijing, China).

### Cloning of araA From *B. adolescentis*

Gene of BAAI (*araA)* was amplified by PCR from the genomic DNA of *B. adolescentis* CICC 6178 using the forward primer P1 (5′-GGGGGATCCACTAGTAGGCCTATG GCAATGGAAAAC-3′) and reverse primer P2 (5′-AGCAGCC AACTGCAGGAGCTCTCAGTGACGGTTGTT-3′) containing the restriction endonuclease sites *Sac*I and *Stu*I (underlined), respectively, and then cloned into pANY1 using the Cloning Kit. Recombinant pANY1-*araA* vector was then transformed into *E. coli* BL21(DE3) competent cells by heat shock method. Transformed strains were grown in LB medium at 37°C for 1 h, and the culture was then transferred to LB plate supplemented with kanamycin (50 μg/mL). Positive colonies were screened by PCR and confirmed by sequencing.

### Phylogenetic Analysis and Multiple Sequence Alignment

Protein sequences of other L-AIs were retrieved from the UniPort. Phylogenetic tree of BAAI was constructed using the neighbor-joining algorithm in MEGA7 (Version 7.0.26). Multiple Sequence Alignment (MSA) of BAAI with structurally characterized L-AIs was done using the Clustal Omega server of EMBL (Sievers et al., 2011), which provided information about conservation of active site residues of L-AIs. The alignment of these proteins was represented by BoxShade 3.21 web server.

### Expression and Purification of Recombinant BAAI

*E. coli* B21(DE3)-*araA* was grown in LB broth at 37°C and 200 rpm until OD_600_ value reached to 0.4–0.6. Then, 1.0 mM of IPTG was added to it for inducing the expression of gene, and incubated for further 10 h at 25°C and 120 rpm (Yun et al., 2018). Due to strong expression and misfolding of BAAI during induction process, recombinant BAAI formed inclusion bodies as also reported for L-AI of *C. hylemonae* (Nguyen et al., 2018). To dissolve inclusion bodies and get BBAI in soluble form, cells were harvested by centrifugation at 8,000 rpm (4°C) for 15 min and washed twice with 100 mM sodium phosphate buffer (pH 7.4) as described earlier (Qi et al., 2016; Zhang et al., 2018). Thereafter, cells were re-suspended in the lysis buffer (50 mM Tris-Cl, 100 mM NaCl, 1% Triton X) and disrupted by sonication (350 W; 1.5 s working time, 2 s interval, for 20 mins, at 4°C), and centrifuged at 10,000 rpm (4°C) for 10 min (Qi et al., 2017). Finally, insoluble BAAI contained in precipitate was dissolved and renatured as per the instruction of the Kit for obtaining soluble and active BAAI for further analysis.

### Quantification of Protein and Determination of Enzyme Activity

Protein concentration was measured by the Bradford method using bovine serum albumin as the standard (Bradford, 1976). Activity of BAAI was determined based on the amount of D-tagatose produced during reaction with D-Galactose under the standard conditions. Reaction mixture consisted of 0.5 mg BAAI enzyme, 0.5 M D-Galactose, and 10 mM MnCl_2_ (pH 7.0). Enzymatic reaction was done at 60°C for 1 h using the method described by Chouayekh et al. (2007). Thereafter, reaction was stopped by adding 0.5 M HCl to the reaction mixture. D-tagatose produced was determined by the cysteine carbazole sulfuric-acid method (Dische and Borenfreund, 2007).

### Determination of Optimum Conditions for Maximum Activity and Stability of BAAI

Experiments were done at various temperatures (45, 50, 55, 58, 60, 62, 65, 68, and 70°C) to determine the optimum temperature for maximum BAAI activity. Thermal stability of BAAI was determined by conducting reactions at 50–80°C for 120 min. Experiments were also carried out under various pH (3–10) conditions at 60°C for 1 h. Target pH was adjusted by using three different buffer systems, namely sodium acetate buffer (pH 3.0–5.0), phosphate buffer (pH 6.0–8.0), and glycine-sodium hydroxide buffer (pH 9.0–10.0). Stability of BAAI at pH was determined by conducting reactions at pH 5–8 for 24 h. Effects of metal ions on the enzyme activity was determined by doing experiments in the presence of various metal ions (FeSO_4_, ZnCl_2_, CoSO_4_, MnCl_2_, NiCl_2_, CaCl_2_, CuCl_2_, EDTA) with 1 mM concentration each. To determine the optimum concentrations of Mn^2+^, reactions were performed under various concentrations of Mn^2+^, ranging from 1 to 10 mM.

### Substrate Specificity and Kinetic Properties of BAAI

Substrate specificity of BAAI was determined under the optimum reaction conditions using 0.5 M of various aldoses (L-arabinose, D-Galactose, D-xylose, D-glucose, and D-mannose). Kinetic parameters of BAAI were determined by conducting reactions in sodium phosphate buffer (100 mM, pH 6.5) at 55°C for 20 min using 10–500 mM of D-Galactose and L-arabinose without adding any metal ions. Kinetic parameters (*K*_m_, *V*_max_, and *K_cat_/K_m_*) were estimated using the non-linear regression software namely GraphPad Prism (Version 6.0, San Diego, CA, United States).

### Structural Homology and Protein-Substrate Docking of BAAI

Homology model was constructed for BAAI with the L-AI of *E. coli* (ECAI) (PDB ID: 4F2D) as a template by SWISS-MODEL (Waterhouse et al., 2018) using default parameters. The Ramachandra plot of model was examined by PROCHECK (Laskowski et al., 1993) to ensure the amino acid residues were not situated in unfavorable region. Protein-substrate docking of BAAI with various substrates were performed using AutoDock Vina software (Trott and Olson, 2009). The interacting amino acid residues of BAAI with ligands were visualized and highlighted using PyMOL software (DeLano, 2002) and Discovery Studio visualizer (Version 17.2.0.16349, San Diego Dassault Systems).

### Bioconversion of D-Galactose Into D-Tagatose

Enzymatic synthesis of D-tagatose from D-Galactose was carried out at 50, 55, 60°C for 10 h. The reaction mixture contained 100 mM of D-Galactose, 6 mM MnCl_2_ and 0.5 mg of the purified enzyme in 100 mM PBS buffer (pH 6.5). Samples were collected every 2 h and D-tagatose was quantified in the high-performance liquid chromatography (HPLC; Shimadzu, GL, Japan; LC-20AT), equipped with a refractive index detector (RID, 40°C) and a Sugar-Ca column (Welch, Shanghai, China; 5mm, 7.8 × 300mm). Column was eluted at 85°C with pure water at a flow rate of 0.7 mL/min.

### Statistical Analysis

Each experiment was repeated in triplicate. Origin 9.0 software (OriginLab Inc., Northampton, MA, United States) was used to perform statistical analysis and data were expressed as mean value ± standard deviation. Data were compared by one-way analysis of variance (ANOVA) considering the least significant difference test (LSD) as *post hoc* test at *p* <0.05 level.

## Results and Conclusion

### Cloning, Sequence Analysis, and Structural Homology of BAAI

**FIGURE 1:**
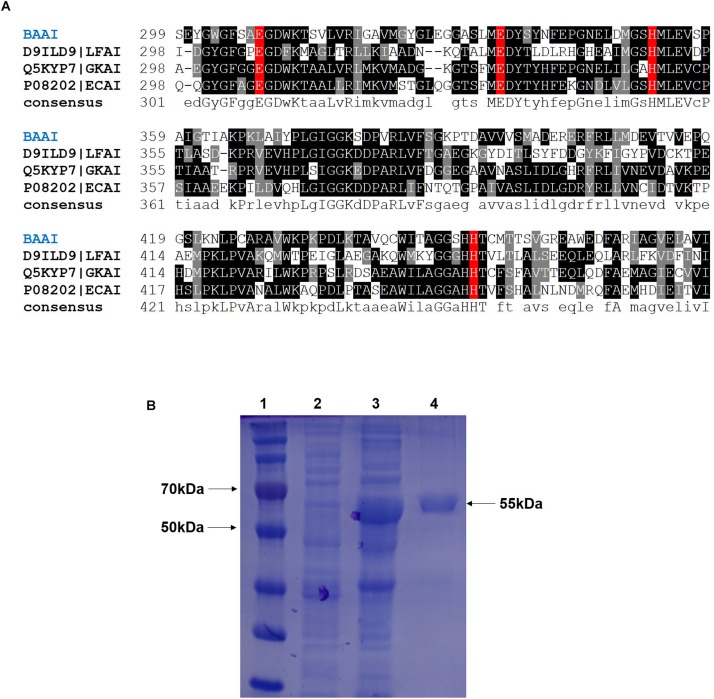
**(A)** Multiple sequence alignment of L-arabinose isomerase (L-AIs) amino acid sequences from *B. adolescentis* (BAAI), *L. fermentum* (LFAI), *G. kaustophilus* (GKAI), *E. coli* (ECAI). The strongly and weakly conserved amino acid residues are indicated by dark and light black, respectively. The amino acids involved in the catalytic activity are shown by the red box. **(B)** SDS-PAGE of the L-arabinose isomerase expressed in *E. coli* BL21(DE3) from *B. adolescentis* CICC 6178 (BAAI). Lane 1, molecular marker; Lane 2, *E. coli* BL21(DE3)/pANY1-*araA* without IPTG induction; Lane 3, *E. coli* BL21(DE3)/pANY1-*araA* with IPTG induction; Lane 4, purified BAAI.

PCR-amplified product of *araA* from *B. adolescentis* was cloned and sequenced. The sequence of the BAAI gene was submitted to the GenBank database (Accession No.: MK929561). The open reading frame (ORF) of *ara*A encoding BAAI was found to be 1515 bp long and encoded a polypeptide with 504 amino acids. LC-MS/MS analysis revealed that molecular weight and isoelectric point of BAAI were 55.85 kDa and 5.06, respectively. The phylogenetic analysis of BAAI with the other L-AIs demonstrated that it had closeness with *B. longum* (I3ZR32), *Thermoanaerobacter mathranii* (Q70G56), and *Thermoanaerobacterium saccharolyticum* (K7SW59). Within the phylogenetic tree, other L-AIs were clustered independently in different groups, while BAAI displayed low identity with those L-AIs. The results of sequence analysis of BAAI using PDB BLAST demonstrated 47% identity with L-AI of *Geobacillus Kaustophilus* (Q5KYP7), 45% with L-AI of *E. coli* (P08202), and 39% with L-AI of *Lactobacillus fermentum* (D9ILD9). Despite amino acid sequence identity of BAAI was slightly higher with L-AI of *G. Kaustophilus* (GKAI) than that found with the L-AI of *E. coli* (ECAI), the latter was used to in the model considering the cofactor (manganese/Mn^2+^), where GKAI did not have the cofactor in its crystal structure. Although the amino acid sequence identity between BAAI with other L-AIs were between 40 and 47%, the MSA of BAAI with the other L-AIs disclosed that amino acid residues at catalytic site were highly conserved. Figure 1A represents MSA of several L-AIs and amino acid residues present in catalytic site (E308, E335, H352, and H452 in BAAI), which were conserved in these L-AIs.

### Expression, Purification, and Activity of BAAI

The cloned and expressed BAAI was purified from inclusion bodies using refolding kit to get the purified and soluble BAAI. Molecular weight of the purified BAAI was around 55 kDa (Figure 1B), which was similar to the molecular weight of other reported L-AIs, like L-AIs of *T. saccharolyticum* (Hung et al., 2014), *L. plantarum* (Chang et al., 2016; Zhang et al., 2020), and *B. longum* (Salonen et al., 2012).

Activity of the purified BAAI was found to be 24.47 U/mg. Previous studies reported varying activities of L-AIs depending on the source strains. Except a high activity of L-AI reported from the pathogenic bacterium, *E. coli* (63 U/mg) (Kim et al., 2001), activity of the most L-AIs reported for other bacteria ranged between 0.44 and 26.4 U/mg (Kim and Oh, 2005; Cheng et al., 2010; Li et al., 2011; Hung et al., 2014; Nguyen et al., 2018). L-AI obtained from *Anoxybacillus flavithermus* showed the highest enzyme activity thus far (26.4 U/mg). However, *A. flavithermus* is a major contaminant of milk powder and gelatin, and hence, it would not be a good source for producing D-tagatose. Therefore, highly active L-AI derived from probiotic strain might have potential for the food-grade D-tagatose production.

### Effects of Temperature and pH on the Enzyme Activity and Stability

**FIGURE 2:**
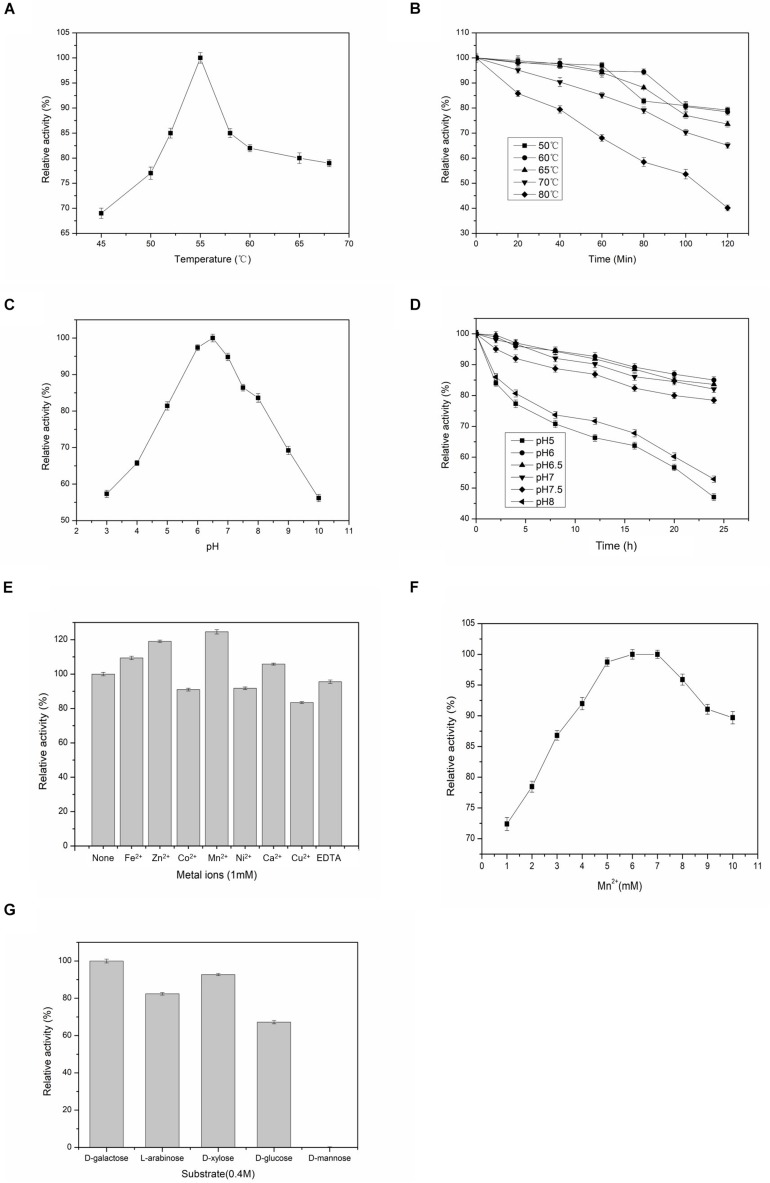
Effects of temperatures, pH, metal ions on the activity and substrate specificities of BAAI. **(A)** activity at various temperature, **(B)** stability at various temperature, **(C)** activity at various pH, and **(D)** stability at various pH. **(E)** Effect of the metal ions on the activity of L-arabinose isomerase of *B. adolescentis* CICC 6178 (BAAI). Activity measured without adding any metallic ion is the control and it represents 100% activity. **(F)** Effects of Mn^2+^ concentrations on the activity of L-arabinose isomerase of *B. adolescentis* CICC 6178 (BAAI). Activities at the optimal Mn^2+^ concentration was defined as 100%. **(G)** Substrate specificities of the L-arabinose isomerase from *B. adolescentis* CICC 6178(BAAI).

Experiments were done at different temperatures ranged between 45 and 70°C to determine the effects of this important factor on the activity of BAAI that varied significantly among the temperatures (*p* < 0.001). As shown in Figure 2A, optimum temperature for the maximum activity of BAAI was found to be 55°C. Similar temperature was also reported as the optimum temperature for L-AI of *B. longum* (Salonen et al., 2012). At 50–65°C, activity of BAAI was stable until 60 min (almost no change), while as much as 80% of the activity retained after 120 min (Figure 2B). On the other hand, a higher temperature above the optimum temperature, particularly at 80°C, activity of BAAI decreased over time (Figure 2B). This might be due to the denaturation of the enzyme at high temperatures and over time, in addition to the generation of unwanted by-products under such conditions. L-AI from the thermophilic and hyper thermophilic microbes, such as L-AI of *Thermotoga maritima*, are highly thermostable and require high temperatures (Lee et al., 2004), which is not viable for the production of D-tagatose on industrial scale. On the other hand, L-AIs from the mesophilic microorganisms, including *L. plantarum* NC8 (Chouayekh et al., 2007) and *L. fermentum* CGMCC2921 (Xu et al., 2011), also showed excellent thermostability, but they were effective at relatively lower temperatures compared to those of thermophilic bacteria. In particular, BAAI showed higher stability than the L-AI of *B. longum* (Salonen et al., 2012).

Effects of pH on the activity of BAAI were determined by conducting experiments at pH 3–10, in which activity varied significantly among the pH conditions (*p* < 0.001). BAAI showed its maximum activity under slightly acidic condition at pH 6.5 (Figure 2C), while more than 90% activity was found at pH 6.0–7.0. Activity of BAAI was stable at a broad range of pH varied from 6.0 to 7.0 until 24 h (Figure 2D). Earlier it was reported that a slightly acidic condition is important for industrial applications of L-AI to minimize by-products formation (Xu et al., 2018). On the other hand, lactose hydrolysis by β-galactosidase for generating D-Galactose was also affected by the acid pH (Zhan et al., 2014). Similar requirement would make the co-production based on β-galactosidase and L-AI from lactose to D-tagatose more efficient. However, most of the reported L-AIs displayed maximal activity of this enzyme at neutral or alkaline pH (Xu et al., 2018). Therefore, thermostable BAAI with optimum activity at a slightly acid pH could hold great potential for D-tagatose production.

### Effects of Metallic Ions on the Activity of BAAI

Activities of most of L-AIs are dependent on divalent metal ions, especially Co^2+^ and Mn^2+^ significantly play important roles on the activity of L-AI (Xu et al., 2018). Compared to the control (without any metal ions), BAAI showed its higher catalytic activity in the presence of Fe^2+^, Zn^2+^, Mn^2+^ (*p* < 0.05), and Ca^2+^, where maximum activity was provided by Mn^2+^ (Figure 2E). On the other hand, Co^2+^, Ni^2+^, Cu^2+^, and EDTA reduced the activity of BAAI to around 80% compared with the activity of the control (Figure 2E). Considering the obtainment of maximum activity with Mn^2+^, experiments were done under various concentrations of this metal ion (Figure 2F). Maximum activity was obtained for 6 mM of Mn^2+^. Above 6 mM of Mn^2+^, enzyme activity was found to be plateaued or even decreased. Possible reasons for such findings could be due to the fact that excessive ions inhibited the enzyme activity to increase the apparent K_m_, which had led to a decrease in the enzyme activity.

### Substrate Specificity of BAAI

L-AIs can bind and act on various aldoses as the substrates. Therefore, substrate specificity of BAAI was determined by conducting experiments with five aldose sugars and results are shown in Figure 2G. Specificity of BAAI varied significantly among the substrates (*p* < 0.01). BAAI showed its maximum specificity to D-Galactose among aldoses tested at a relative activity of 100%, while no conversion was observed for D-mannose. Specificities of BAAI to the other three substrates were found to be 67.2% for D-glucose, 82.4% for L-arabinose, and 92.7% for D-xylose. Earlier, almost all of the reported L-AIs showed specificities with both L-arabinose and D-Galactose, in which higher specificities were recorded with L-arabinose than D-Galactose (Xu et al., 2018). For example, L-AIs of *Acidothermus cellulolytics* ATCC 43068 (Cheng et al., 2010), *Bacillus stearothermophilus* US100 (Rhimi and Bejar, 2006), and *Lactobacillus reuteri* (Petra et al., 2014) showed maximum specificities with L-arabinose. Specially, L-AIs of *B. subtilis* str. 168 (Kim et al., 2010) and *Pseudoalteromonas haloplanktis* ATCC 14393 (Xu et al., 2016) showed specificity only to L-arabinose and could not convert D-Galactose into D-tagatose.

### Kinetic Parameters of BAAI

Non-linear regression fitting of Michaelis–Menten equation was determined for two major substrates, namely D-Galactose and L-arabinose under the optimum conditions (55°C, pH 6.5). Initial velocities were determined in the standard assay mixture. D-Galactose and L-arabinose had hyperbolic saturation curves, and the corresponding double-reciprocal plot was linear. The *K*_m_ values of BAAI for D-Galactose and L-arabinose were 22.4 mM (*V*_max_, 489 U/mg) and 40.2 mM (*V*_max_, 275.1 U/mg), respectively. Interestingly, *K*_m_ for L-arabinose was almost twofolds higher than that of D-Galactose, while *V*_max_ was twofolds lower for the former than for the latter. The *K_cat_/K_m_* values for D-Galactose and L-arabinose were found to be 9.3 mM^–1^ min^–1^ and 8.6 mM^–1^ min^–1^, respectively. The higher *V*_max_, and *K_cat_/K_m_* of BAAI with D-Galactose indicated a higher catalytic efficiency of BAAI with D-Galactose, which were compared and summarize in Table 1 with other L-AIs.

**TABLE 1:** Comparative kinetic characteristics of L-AIs obtained from various microbial species determined for D-Galactose and L-arabinose as the substrates.

Microorganisms	Temperature (°C)	pH	Metal ions	*K*_m_ (mM)		*K_cat_/K_m_* (mM^–1^ min^–1^)		References
				
				D-Gal	L-Ara	D-Gal	L-Ara	
*A. Flavithermus*	95	9.5–10.5	Ni^2+^	25.2	78.5	5.2	0.67	Li et al., 2011
*G. stearothemophilus* DSM 22	70	7.0–7.5	Mn^2+^, Mg^2+^	117	63	4.3	32.5	Lee et al., 2005
*G. kaustophilus*	60	7.5	Mg^2+^, Ca^2+^	86.1	11.3	NR	NR	Choi et al., 2016
*G. thermodenitrificans*	70	8.5	Mn^2+^	408	142	0.5	48	Kim and Oh, 2005
*B. subtilis* str. 168	32	7.5	Mn^2+^	NR	NR	NR	121	Kim et al., 2010
*E. coli*	40	8.0	Mn^2+^, Fe^2+^	NR	NR	NR	NR	Yoon et al., 2003
*B. longum* NRRL B-41409	55	6.0–6.5	Ca^2+^, Mg^2+^	590	120	0.72	48	Salonen et al., 2012
*T. mathranii* DSM 11426	65	8.0	Mn^2+^	120	80	NR	NR	Jørgensen et al., 2004
*T. saccharolyticun* NT0U1	70	7.0–7.5	Mn^2+^, Co^2+^	122	NR	2.41	NR	Hung et al., 2014
*B. licheniforus* ATCC 14580	50	7.5	Mn^2+^	NR	369	NR	34	Prabhu et al., 2008
*Pediococcus pentosaceus* PC-5	50	6.0	Mn^2+^, Co^2+^	66	NR	2.9	NR	Men et al., 2014
*L. fermentum* CGMCC 2921	65	6.5	Mn^2+^, Co^2+^	60	NR	9.0	19	Xu et al., 2011
*L. plantarum* NC8	60	7.5	Mn^2+^	69.7	43.4	1.6	15.5	Chouayekh et al., 2007
*B. adolenscentis* CICC 6178	55	6.5	Mn^2+^, Zn^2+^	22.4	40.2	9.3	8.6	This study

*NR, Not Reported.*

### Structural Homology and Protein-Substrate Docking

**FIGURE 3:**
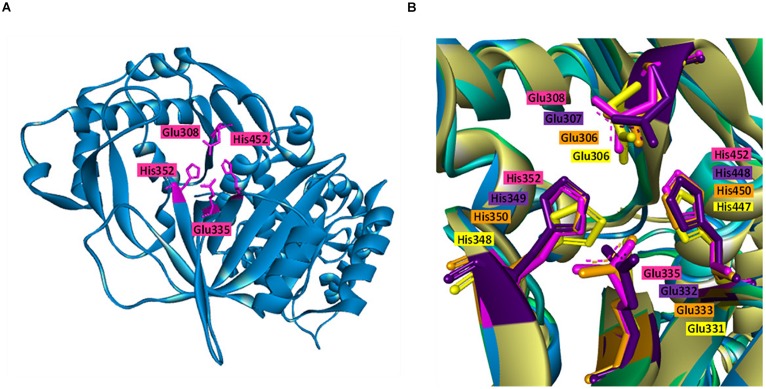
Homology model and catalytic pocket of L-AI. **(A)** Homology model of L-AI of *B. adolescentis* CICC 6178 (BAAI) with the catalytic residues (shown in pink colure). **(B)** Superimposition of the catalytic residues of BAAI (pink), LFAI (violet), ECAI (orange), and GKAI (yellow), showing orientation of the catalytic residues.

Biochemical and sequence analyses of BAAI displayed substrate specificity with four different type of substrates and conservation of catalytic active site with other L-AIs. For further analysis of substrates interaction and structural compression, homology model was generated and docked with the substrates. Homology model of BAAI was generated using ECAI (4F2D) as a template by SWISS-MODEL (Figure 3A). This model contained 99.8% of the total amino acid residues in the favorable and allowed regions, whereas only 0.2% of residues in the disallowed regions. MSA analysis of the protein revealed that high conservation was located at the catalytic sites, which was structurally superimposed as determined by the discovery studio visualizer (Figure 3B). The superimposed structures of the proteins showed similar orientation of the catalytic amino acids (Figure 3B).

**FIGURE 4:**
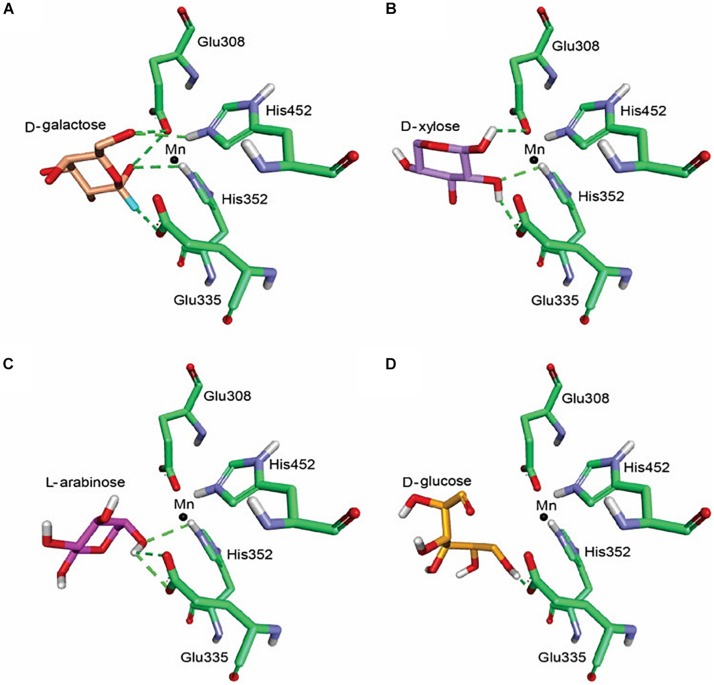
Molecular docking of BAAI for the active sites that interact with D-Galactose **(A)**, D-xylose **(B)**, L-arabinose **(C)**, and D-glucose **(D)**. The green dotted line denotes the H-bonds between the substrates and the active sites of BAAI.

BAAI demonstrated substrate specificities with D-Galactose, D-xylose, L-arabinose, and D-glucose in the biochemical assay (Figure 2G). Further investigation on the interaction of BAAI with these substrates were revealed by docking with 3-D homology model. Among the substrates, D-Galactose exhibited interaction with all four catalytic residues through five H-bonds (Figure 4A), whereas D-xylose and L-arabinose showed three H-bonds (Figures 4B,C). On the other hand, D-glucose could bind loosely with the active site of BAAI due to a single H-bond (Figure 4D). These results could explain the reasons for varying specificity of BAAI to different substrates that were solely for the difference in the interaction of these substrates with BAAI. Based on the biochemical and docking analyses, D-Galactose would be the best substrate for BAAI, followed by D-xylose, L-arabinose, and D-glucose.

### Enzymatic Conversion for D-Tagatose Production

**FIGURE 5:**
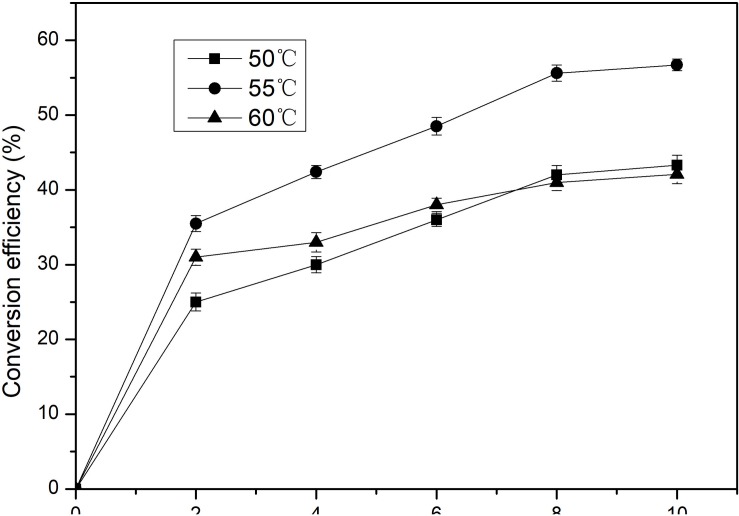
Conversion of D-Galactose to D-tagatose by the purified BAAI over time at different temperatures.

Isomerization reaction of D-Galactose to D-tagatose by BAAI was further investigated at different temperature (50, 55, and 60°C) and pH 6.5 for 10 h. The maximum conversion of D-Galactose into D-tagatose reached after 8 h (Figure 5). Conversion efficiency of D-Galactose to D-tagatose reaction were nearly 40% at 50 and 60°C, which was 56.7% at 55°C. Conversion efficiency increased with time at 55°C and reached to the maximum after 8 h. This maximum conversion efficiency of BAAI was higher than the efficiency reported for several other L-AIs earlier, such as L-AI of *L. plantarum* NC8 (30%, 6 h, at 60°C) (Chouayekh et al., 2007), and L-AI from *A. cellulolytics* (53%, 12 h at 75°C) (Cheng et al., 2010). This study showed that the probiotic-derived BAAI has good potential for the production of D-tagatose, which suggested that the intestinal bacteria *Bifidobacterium* and lactic acid bacteria, could be good sources of L-AI.

## Conclusion

This study elucidates the biochemical and structural features of BAAI for the first time, with revealing its efficiency to convert D-Galactose into D-tagatose. The comparative structural analysis of the characterized BAAI and other L-AIs had similar amino acid residues conservation at the catalytic sites. The protein-ligand docking of BAAI identified a strong interaction between the residues of catalytic site and D-Galactose (preferred substrate). Subsequent bioconversion of D-Galactose into D-tagatose with the purified BAAI provided 56.7% conversion efficiency after 10 h at 55°C, which is excellently comparable with the efficiencies of other L-AIs reported thus far.

## Data Availability Statement

All datasets generated for this study are included in the article/supplementary material.

## Author Contributions

XQ, GZ, and YA designed the study. GZ performed all the experiments, analyzed the data, and drafted the manuscript. AP assisted in the docking studies. JY assisted in the recombinant strain construction and protein purification. HZ and XQ helped to revise the manuscript. All authors have read and approved the manuscript.

## Conflict of Interest

The authors declare that the research was conducted in the absence of any commercial or financial relationships that could be construed as a potential conflict of interest.
